# Lipidomics Workflow for Analyzing Lipid Profiles Using Multiple Reaction Monitoring (MRM) in Liver Homogenate of Mice with Non-alcoholic Steatohepatitis (NASH)

**DOI:** 10.21769/BioProtoc.4773

**Published:** 2023-07-05

**Authors:** Hai Ning Wee, Lye Siang Lee, Sharon Hong Yu Han, Jin Zhou, Paul Michael Yen, Jianhong Ching

**Affiliations:** 1 Cardiovascular and Metabolic Disorders Programme, Duke-NUS Medical School, Singapore, Singapore; 2 KK Research Centre, KK Women’s and Children’s Hospital, Singapore, Singapore

**Keywords:** Lipidomics, Liver, LC-MS, Sphingolipids, Fatty acids, Glycerolipids, Phospholipids, Cholesteryl esters

## Abstract

Non-alcoholic steatohepatitis (NASH) is a condition characterized by inflammation and hepatic injury/fibrosis caused by the accumulation of ectopic fats in the liver. Recent advances in lipidomics have allowed the identification and characterization of lipid species and have revealed signature patterns of various diseases. Here, we describe a lipidomics workflow to assess the lipid profiles of liver homogenates taken from a NASH mouse model. The protocol described below was used to extract and analyze the metabolites from the livers of mice with NASH by liquid chromatography–mass spectrometry (LC-MS); however, it can be applied to other tissue homogenate samples. Using this method, over 1,000 species of lipids from five classes can be analyzed in a single run on the LC-MS. Also, partial elucidation of the identity of neutral lipid (triacylglycerides and diacylglycerides) aliphatic chains can be performed with this simple LC-MS setup.

Key features

Over 1,000 lipid species (sphingolipids, cholesteryl esters, neutral lipids, phospholipids, fatty acids) are analyzed in one run.

Analysis of liver lipids in non-alcoholic steatohepatitis (NASH) mouse model.

Normal-phase chromatography coupled to a triple quadrupole mass spectrometer.

**Graphical overview**


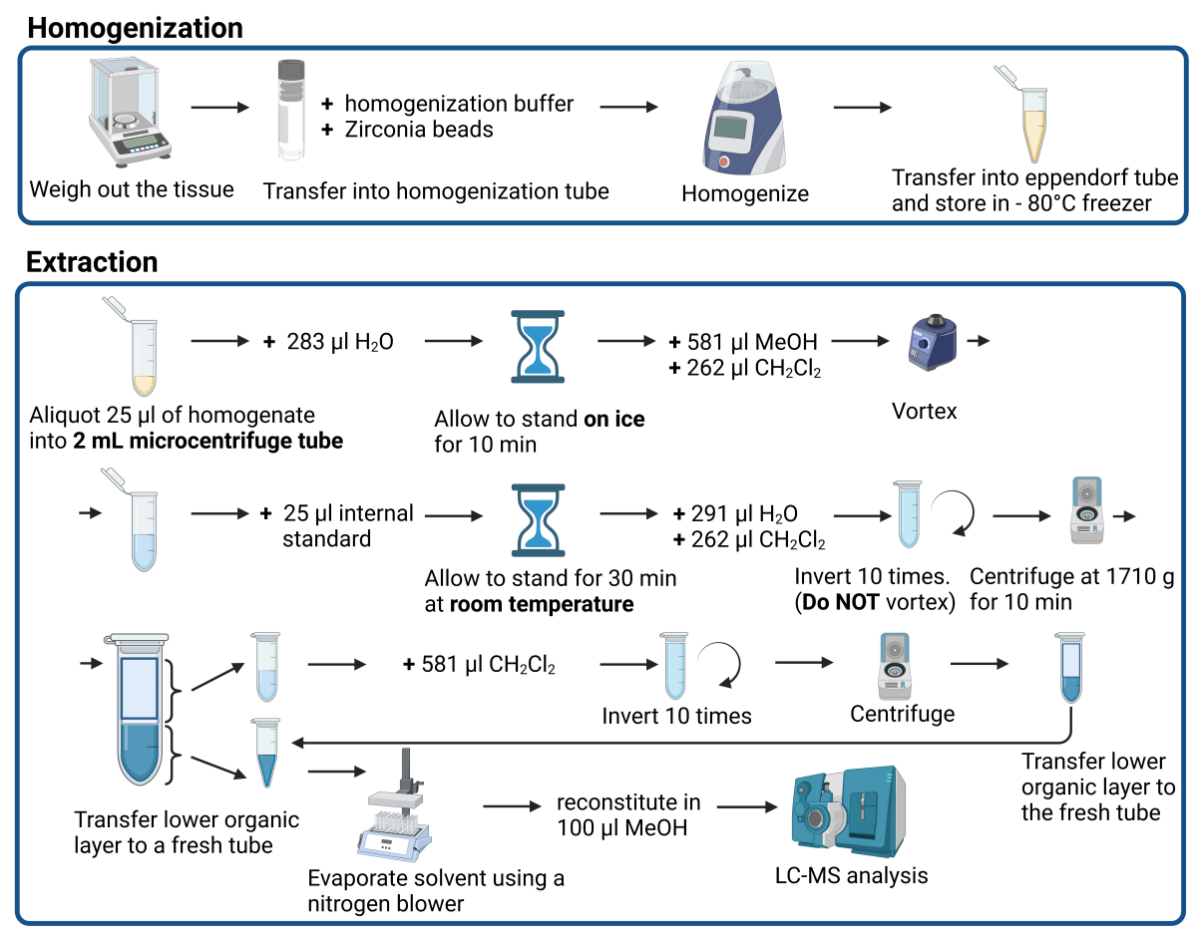


**Schematic procedure for the homogenization and extraction of mouse liver tissue in preparation for LC-MS analysis (Created with** **BioRender.com**)

## Background

Lipids are essential components of energy metabolism that can be disrupted in metabolic diseases. Lipids comprise various classes, each possessing widely different chemical properties, e.g., steroids are much more non-polar than phospholipids. Due to the widely differing properties, it is difficult to have a single method catered to all the different lipids. As a result, different classes of lipids often have dedicated analytical methods, such as various derivatization techniques, columns, and mass spectrometers, which makes it challenging to have an overview of a broad spectrum of lipids in a single analysis (Wu et al., 2020; Zhao et al., 2020). Another issue of lipid analysis pertains to the triglyceride class. With up to three aliphatic chains attached to a glycerol backbone, the task of identifying and quantifying all species is highly challenging and has been extensively discussed, with each technique having its own limitations and advantages (Han and Ye, 2021). Compared to other methods requiring adaptations, such as two-dimensional LC, argentation, or supercritical fluid chromatography, the method described here provides a broad view of various lipid classes using standard methods that can be done in most laboratories with basic training.

**Figure 1.**
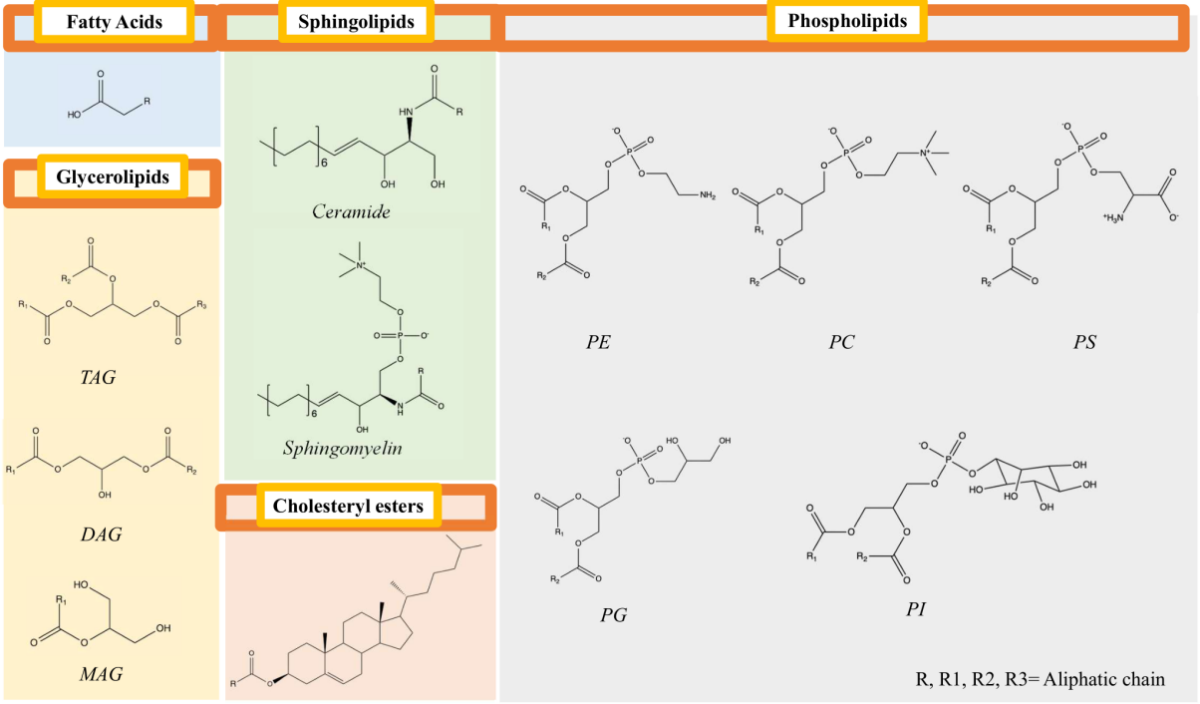
Key structural features of five broad categories of lipids are included in the lipidomics panel.

In this protocol, we describe one variant of a lipidomics workflow, originally developed by the mass spectrometry company SCIEX, that covers over five classes of lipids (sphingolipids, phospholipids, glycerolipids, cholesteryl esters, and fatty acids) (Figure 1), including a partial identification of a single aliphatic chain within the neutral lipid, triacylglycerol (Ubhi et al., 2016). The workflow uses a variety of lipid standards, including stable isotopes from ceramides, sphingomyelins, cholesteryl esters, phosphatidylcholines, phosphatidylethanolamines, phosphatidylglycerols, phosphatidylinositols, phosphatidylserines, monoacylglycerols, diacylglycerols, and triacylglycerols. The system uses a normal-phased hydrophilic interaction chromatography (HILIC) column, which separates the lipids by classes as opposed to chain length in reverse-phased systems. In total, the multiple reaction monitoring (MRM) method performs a single-point quantitation of over 1,000 lipid species, providing a convenient overview of common and important lipids for studying metabolic diseases. Here, we apply the techniques to studying the lipidome of liver tissue from a mouse model with non-alcoholic steatohepatitis (NASH). These techniques are not limited to studying the liver but can be applied to other tissues, including cell culture.

**Part I: Tissue homogenization**

## Materials and reagents

1. 2 mL cryogenic vial (Corning, catalog number: 430488)
2. 2 mL homogenization tubes (Labcon Co., catalog number: 3661-875-000)
3. Zirconia beads, 1.0 mm diameter (BioSpec Products, catalog number: 11079110ZX)
4. 0.9% sodium chloride solution (Sigma-Aldrich, catalog number: S8776)
5. Cylinder of liquid nitrogen
6. Acetonitrile Optima^®^, LC-MS grade (Fisher chemical, catalog number: A955-4)
7. Deionized (MilliQ) water (Arium Pro System, Sartorius AG)
8. Formic acid, HPLC grade (Merck Supelco, catalog number: 5.43804)
9. Dry ice
10. Homogenization buffer (see Recipes)

## Recipes

1. **Homogenization buffer**
  **Table:** 

	Final concentration	Amount
Acetonitrile	n/a	498.5 mL
Deionized H_2_O	n/a	498.5 mL
Formic acid	0.3%	3 mL

## Equipment

1. Precellys Evolution tissue homogenizer (Bertin Technologies, France)
2. Cryolys Evolution cooling unit (Bertin Technologies, France)

## Procedure

1. Pre-chill 0.9% sodium chloride solution on wet ice before animal procedures.
2. Euthanize the mouse using an approved protocol and remove the liver within 10 min. Wash the liver in cold sodium chloride solution to remove blood on the organ’s surface. Cut the liver into smaller pieces before keeping it in the cryogenic vials, which are snap frozen immediately in liquid nitrogen. The tubes of snap-frozen liver samples can be kept in a -80 °C freezer until ready to be used.
3. Accurately weigh 50–60 mg of frozen liver tissue samples into 2 mL homogenization tubes and immediately place them on wet ice.
4. Perform all following procedures on wet ice, unless otherwise stated.
5. Prepare homogenization buffer (see Recipes).
6. Add ice-cold homogenization buffer into each homogenization tube to obtain a sample concentration of 50 mg/mL, after assuming a water content of 50 μL in 50 mg of tissue.
7. Add Zirconia beads with a diameter of 1.0 mm to approximately 25% of the buffer level.
8. Place the dry ice, the homogenizing tubes containing the liver tissues, the homogenizing buffer, and Zirconia beads in a Precellys Evolution tissue homogenizer equipped with a Cryolys Evolution cooling unit for six cycles of 20 s at 6,500 rpm at 4 °C, with 10 s pause intervals to minimize temperature fluctuations during homogenization.
9. Aliquot 50 μL of the homogenate and snap freeze in dry ice before storing at -80 °C until further sample processing.

**Part II: Lipidomics analysis using a triple quadrupole mass spectrometer**

The lipidomics analysis was performed using the protocol provided by SCIEX, as described in the application notes (Ubhi et al., 2016; Ubhi, 2020; Pearson et al., 2023). We selected this protocol because it has been used in many studies and demonstrated good reproducibility (Cao et al., 2019; Loef et al., 2020), accuracy, and precision (Contrepois et al., 2018). The SCIEX Lipidyzer^TM^ platform was developed to provide MS-based targeted profiling of over 1,000 lipid species spanning 13 lipid subclasses. The > 1,000 lipid analytes included in this lipidomics method fall into five broad categories (Figure 1), based on their basic structural units as described below:

1. **Glycerolipids** consist of a glycerol backbone with fatty acids attached to it by ester linkage. They include mono-, di-, and triglycerides (MAG, DAG, TAG).
2. **Sphingolipids** consist of a sphingoid long-chain backbone (normally sphingosine or sphinganine) with a fatty acid attached to the C2 position by an amide bond and another moiety (i.e., H, sugar, or phospho-X group) attached at C3. The biologically relevant subclasses include ceramides (CER), dihydroceramides (DCER), lactosylceramides (LCER), hexosylceramides (HCER), and sphingomyelins (SM). SM is also considered a glycerophospholipid.
3. **Glycerophospholipids** consist of a glycerol backbone with fatty acids attached to the first two carbon atoms (C1 and C2), while a polar phospho-X headgroup is attached to the C3 position. They include phosphatidylcholine (PC), phosphatidylethanolamine (PE), phosphatidylglycerol (PG), phosphatidylinositol (PI), and phosphatidylserine (PS). Lysophospholipids are glycerophospholipids lacking one acyl chain. Hence, their glycerol unit is attached to one fatty acid and one polar phospho-X headgroup. They include lysophosphatidylcholine (LPC), lysophosphatidylethanolamine (LPE), lysophosphatidylglycerol (LPG), lysophosphatidylinositol (LPI), and lysophosphatidylserine (LPS).
4. **Free fatty acids (FA)** include acyl chains of various lengths, such as palmitic acid (FA 16:0).
5. **Cholesterol esters (CE)** consist of cholesterol with a long-chain fatty acid esterified to its hydroxyl group.
  One of the major obstacles that curtail the absolute quantification in MS-based lipidomics method is the matrix effects, whereby the components that co-elute with the analyte of interest may influence its ionization efficiency (Köfeler et al., 2021; Swinnen and Dehairs, 2022). For instance, matrix effects have been known to cause significant ion suppression of phospholipids, e.g., phosphatidylcholine (Guo and Lankmayr, 2011; Khoury et al., 2016) and lysophosphatidylcholine (Morita et al., 2019), in electrospray ionization (ESI)-based methods, making exact quantification difficult. To partially compensate for the influence of matrix effects, we have included an internal standard (IS) corresponding to each of the following lipid classes: SM, CE, CER, DCER, HCER, LCER, TAG, LPC, PC, LPE, PE, and FA. These internal standards also serve to correct for any possible variations during the process of sample preparation and analysis (Wang et al., 2016). Given the large number of lipid analytes (>1,000) in this panel, it is not always possible to find an appropriate internal standard for each lipid class. For lipid classes with a matching internal standard, concentrations of the lipids in samples can be quantified using a single-point quantitation or isotope dilution method. However, for lipid classes where a matching stable isotope internal standard is unavailable, we select one of the internal standards from a different lipid class as its surrogate internal standard and express its concentration as an area-ratio of the analyte to the surrogate internal standard. Therefore, the final data provided by this lipidomics panel should be regarded as relative quantification for lipid species with surrogate internal standards.
**Extraction method**
  The extraction method is based on the protocol developed by SCIEX. Essentially, this method involves the partitioning of lipids in a biphasic mixture of dichloromethane (CH_2_Cl_2_) and methanol. We made a slight modification to the volume of extraction solvents used, so that the final volume could fit within a 2 mL microcentrifuge tube. Since the lipid-containing organic phase lies at the bottom of the tube, one must exercise caution when collecting the organic phase using a micropipette (Wong et al., 2019). It is advisable to use micropipette tips of 200 μL or smaller when performing this procedure, so it will be easier to pass through the overlying aqueous phase and non-extractable residue layer to reach the organic phase at the bottom. The detailed protocol for extraction is described below.

## Materials and reagents

1. 2 mL microcentrifuge tubes (Corning Axygen^®^, catalog number: MCT-200-C)
2. Waters XBridge Amide column 4.6 mm × 150 mm, 3.5 μm (Waters Corp, catalog number: 186004869)
3. Dichloromethane (CH_2_Cl_2_) (Sigma-Aldrich, catalog number: 270997)
4. Methanol Optima^®^ (MeOH) (Fisher Chemical, catalog number: A456-4)
5. Ammonium acetate Bioxtra > 98% (Sigma-Aldrich, catalog number: A7330-100g)
6. Ammonium hydroxide solution (Sigma-Aldrich, catalog number:338818)
7. Acetonitrile Optima^®^ (Fisher Chemical, catalog number: A955-4)
8. Internal Standards kit for Lipidyzer^TM^ platform (SCIEX, catalog number: 5040156)
9. Deionized (MilliQ) water (Arium Pro System, Sartorius AG, Germany)
10. Liquid chromatography solvent A
11. Liquid chromatography solvent B
12. Nitrogen cylinder
13. Internal Standards kit for Lipidyzer^TM^ Platform (see Recipes)
14. Liquid chromatography solvent A (pH 8.2, adjusted using ammonium hydroxide) (see Recipes)
15. Liquid chromatography solvent B (pH 8.2, adjusted using ammonium hydroxide) (see Recipes)

## Recipes

1. **Internal Standards kit for Lipidyzer^TM^ Platform**
  Store standards as per manufacturer’s instructions in a freezer at -25 °C; standards need to be pre-mixed as per the table below.
  **Table:** 

Internal standard	Stock concentration (mg/mL)	Volume to mix (μL)	Final concentration (μM)
CER(16:0) (d9)	0.02	110	36.57
CE(22:6) (d7)	0.15	110	213.18
TAG(52:1/FA18:0) (d9)	0.14	109	160.95
DCER(16:0) (d9)	0.004	110	7.29
FFA(17:1)	0.05	110	186.23
HCER(16:0) (d9)	0.03	110	42.31
LCER(16:0) (d9)	0.03	110	34.43
LPC(16:0) (d9)	0.1	110	198.14
LPE(18:0) (d5)	0.05	110	102.75
PC(16:0/16:1) (d9)	0.0625	76	84.39
PE(18:0/18:1) (d5)	0.01	109	13.32
SM(16:0) (d7)	0.1	110	140.92
2. **Liquid chromatography solvent A (pH 8.2, adjusted using ammonium hydroxide)**
  **Table:** 

Reagent	Final concentration	Amount
Ammonium acetate	1 mM	n/a
Deionized H_2_O	n/a	50 mL
Acetonitrile	n/a	950 mL
3. **Liquid chromatography solvent B (pH 8.2, adjusted using ammonium hydroxide)**
  **Table:** 

Reagent	Final concentration	Amount
Ammonium acetate	1 mM	n/a
Deionized H_2_O	n/a	500 mL
Acetonitrile	n/a	500 mL

## Equipment

1. Sorvall Legend Micro 21R microcentrifuge (Thermo Fisher Scientific., catalog number: 75002447)
2. Vortex-Genie 2 vortex mixer (Scientific Industries, catalog number: SI-0236)
3. SCIEX Triple Quad 5500 MS System
4. Agilent 1290 Infinity II LC System

## Software

1. Multi-Quant software (SCIEX)

## Procedure

**Quality control samples**

1. Ensure that appropriate controls are in place prior to extracting the samples. Required controls include:
  - Blank solution without internal standards (methanol vehicle only).
  - Blank solution with internal standards (75 μL of methanol vehicle spiked with 25 μL of internal standard mix).
  - Pooled quality control samples, made by pooling at least 10% of samples in a large batch study, or an equal volume of each sample into a single tube. These pooled quality control replicates are run regularly over the entire batch, so that any variations can be monitored across the entire run. This is especially important for large study samples that are performed over multiple days.

**Extraction procedure**

1. Thaw liver homogenates atop ice; then, pipette 25 μL of each liver homogenate to a 2 mL microcentrifuge tube.
2. Add 283 μL of deionized H_2_O to 25 μL of liver homogenate and allow the sample to stand on ice for 10 min.
3. Pipette 581 μL of MeOH to the mixture.
4. Pipette 262 μL of CH_2_Cl_2_ to the mixture.
5. Cap the tube and vortex the mixture vigorously for 5 s at maximum speed.
6. Ensure that the mixture consists of only one single phase. If two distinct phases are observed, add 25 μL of MeOH and vortex, and then check to ensure that a single phase is formed. Otherwise, continue adding 25 μL of MeOH and vortex until a single phase is seen.
7. Add 25 μL of the premixed lipid internal standard, then vortex and incubate the mixture for 30 min at room temperature.
8. Pipette 291 μL of deionized H_2_O to the mixture.
9. Pipette 262 μL of CH_2_Cl_2_ to the mixture.
10. Gently invert tubes 10 times. DO NOT VORTEX the tube; otherwise, an emulsion will be formed (Video 1).
  **Video 1.**
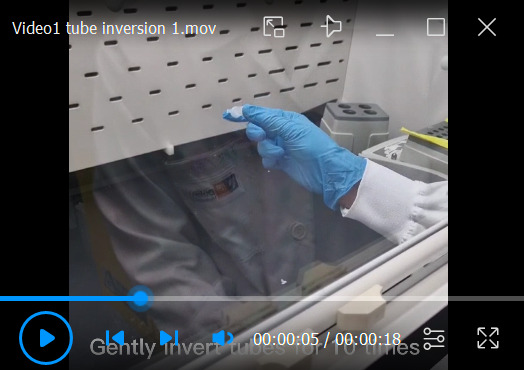
Inversion of tube
11. Pre-chill centrifuge to 4 °C. Subject the tube to centrifugation at 1,710× *g* for 10 min.
12. Transfer the lower organic layer to a fresh microcentrifuge tube (Video 2).
  **Video 2.**
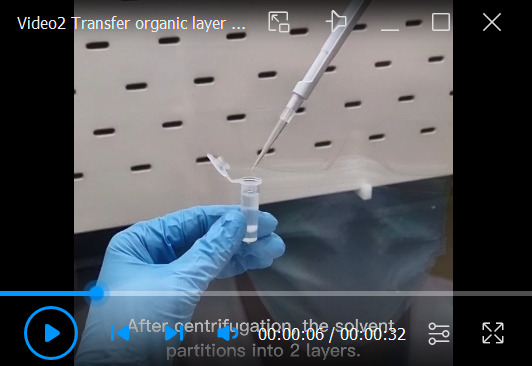
Transfer of organic layer
13. Pipette 581 μL of CH_2_Cl_2_ to the remaining extracts in the tube.
14. Mix by gently inverting 10 times, centrifuge, and then transfer the lower organic layer to the tube from step 12.
15. Evaporate solvent using a nitrogen blower.
16. Reconstitute the extracted lipids in 100 μL of MeOH.

**LC method and parameters**

Chromatographic separation of lipid classes is achieved using a normal-phase column by gradient elution of a 24-min timeframe. The elution order of solutes in normal-phase separation is governed by polarity, with solutes of lower polarity eluting first. Therefore, as a general rule, lipid species within each subclass elute in the order of decreasing chain length (i.e., longer chains elute first) and increasing degree of unsaturation of the fatty acyl groups (i.e., species with more double bonds elute later). The stationary phase (HILIC column) and mobile phases (A and B) are described below:

• Stationary phase: XBridge Amide 3.5 μm, 4.6 × 150 mm column.

• Mobile phase A: 1 mM ammonium acetate in 95% acetonitrile.

• Mobile phase B: 1 mM ammonium acetate in 50% acetonitrile (pH to be adjusted by adding ammonium hydroxide).

One should remember to add ammonium hydroxide to adjust the pH of mobile phase B to match the pH of mobile phase A. This is an important step because the retention times on a HILIC column are very sensitive to the pH changes in the mobile phase.

The LC parameters are shown below:

• Gradient

**Table:** 

Time (min)	Flow rate (mL/min)	Solvent A	Solvent B
0	0.7	94	6
6	0.7	94	6
10	0.7	75	25
11	0.7	2	98
13	0.7	0	100
13.4	0.7	0	100
13.5	1.5	0	100
18.6	1.5	0	100
18.7	1.5	99.9	0.1
23	1.5	99.9	0.1
23.5	0.7	99.9	0.1
24	Stop		

• Oven temperature = 35 °C

**MS method and parameters**

The method utilizes MRM quantification approach in a triple quadrupole LC-MS instrument to ensure high selectivity of lipid species. Positive ion mode is used to detect SM/CER/DCER/HCER/LCER/TAG/DAG/MAG, while negative ion mode is used to detect PC/PE/PG/PI/PS/FFA.

In MRM, the lipids that have been ionized are scanned in Q1, fragmented to produce product ions in Q2 (also known as the collision cell), and these product ions are scanned in Q3. The first (Q1) and the last (Q3) mass analyzers of the triple quadrupole instrument are used as mass filters to isolate a precursor ion and a corresponding product ion for each lipid species. Generally, the product ions included in this method are either characteristic of the headgroup or the backbone of each lipid class, or they are specific for one of the fatty acids attached to the lipid species. For example, sphingomyelins undergo fragmentation to yield a phosphorylcholine head group as its product ion at *m/z* 184 (Chen et al., 2010). Ceramides other than sphingomyelins fragment at their sphingoid base backbones to produce either sphingosine at *m/z* 264 (CER, HCER) or sphinganine at *m/z* 266 (e.g., DCER, LCER) as their product ions (Chen et al., 2010). Cholesterol esters yield the cholesterol moiety as its product ion at *m/z* 369.4 (Yu et al., 2014). The phospholipids (PC, PE, PG, PI, PS, LPC, LPE, LPG, LPI, and LPS) fragment to yield one of the fatty acid chains as its product ion (Pi et al., 2016). Fragmentation of glycerolipids (e.g., TAG, DAG, MAG) produces a neutral loss corresponding to one of the fatty acyl chains.

Besides Q1 and Q3 transitions, the mass spectrometry method has to be optimized for collision energy and other parameters. Table A1 in the appendix shows the MRM parameters for each of the lipid metabolites. These parameters will need to be re-optimized in different triple quadrupole instruments.

**Figure 2.**
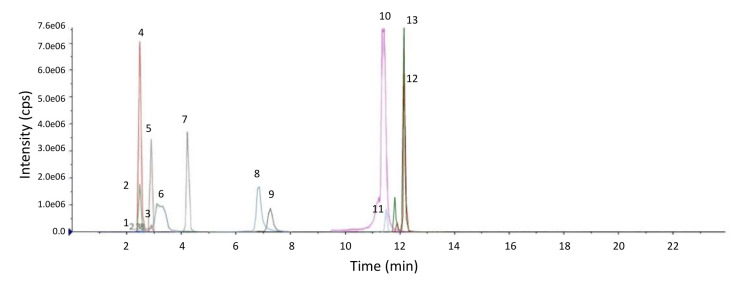
Chromatogram of lipid internal standards. 1: CE(22:6) (d7); 2: CER(16:0) (d9); 3: DCER(16:0) (d9); 4: FFA(16:0) (d9); 5: FFA(17:1); 6: TAG(52:1_FA18:0) (d9); 7: HCER(16:0) (d9); 8: PE(18:0_18:1) (d5); 9: PC(16:0_16:1) (d9); 10: SM(16:0) (d7); 11: LCER(16:0) (d9); 12: LPE(18:0) (d5); 13: LPC(16:0) (d9).

The MRM scans are scheduled, which means that the instrument only scans for each Q1/Q3 transitions at a specific window, corresponding to the time when the particular lipid class elutes. Therefore, it is important to ensure that the MS peak of interest does not drift out of the scan window as a result of retention time shifts (usually due to changes in mobile-phase composition or column aging). Thus, every time after column or solvent changeovers, it is important to check the retention time to ensure that accurate and appropriate scan windows are set for each lipid of interest. The importance of this is highlighted in other lipidomics protocols as well (Mukhamedova et al., 2020). A typical chromatogram of the representative lipid standards is shown in Figure 2.

## Data analysis

**Data integration and analysis**

Each peak area is integrated using Multi-Quant software. Default settings of peak width, peak height, noise, baseline, and peak splitting on Multi-Quant should generally be able to screen peaks with reasonable quality for integration. Changes to default values should be done with care if optimization is required. A clear and easy reference for using Multi-Quant can be found on https://www.youtube.com/watch?v=yjNvcf9CEL0, an instructional video created by SCIEX. After peak integration, peak areas and the area ratios to internal standards can be exported from the software into Microsoft Excel table format for further processing. To determine the relative concentration of each lipid, the area of each lipid of interest is divided by the area of the relevant internal standard. Normalized fold-change can be computed for each lipid species by dividing the area-ratio of each sample by the mean area-ratio across all the samples. A heatmap can be generated using the normalized fold-change values. It is generally accepted that at least three to five biological replicates should be tested in order to get reasonable data for statistical analysis.

For lipid species with the corresponding internal standards (i.e., SM, CE, CER, DCER, HCER, LCER, TAG, LPC, PC, LPE, PE, and FA), the concentration can be computed by dividing the area of peak by the area of the internal standard and multiplying by the final concentration of internal standard in the sample (Table 1). Detailed application and statistical analysis of the lipidomics data can be found in the statistical analysis section of Zhou et al. (2022), where this protocol was originally described.

**Table 1.** Lipid classes and their respective internal standard for normalization

Lipid class	Internal standard for normalization
SM	SM (16:0) (d7)
CE	CE(22:6) (d7)
CER	CER(16:0) (d9)
DCER	DCER(16:0) (d9)
HCER	HCER(16:0) (d9)
LCER	LCER(16:0) (d9)
TAG	TAG(52:1/FA18:0) (d9)
DAG	TAG(52:1/FA18:0) (d9)
MAG	TAG(52:1/FA18:0) (d9)
LPC	LPC(16:0) (d9)
PC	PC(16:0/16:1) (d9)
LPE	LPE(18:0) (d5)
PE	PE(18:0/18:1) (d5)
LPG	LPC(16:0) (d9)
PG	PC(16:0/16:1) (d9)
LPI	LPC(16:0) (d9)
PI	PC(16:0/16:1) (d9)
LPS	LPC(16:0) (d9)
PS	PC(16:0/16:1) (d9)
Myristic acid	FFA(17:1)
Palmitic acid	FFA(17:1)
Stearic acid	FFA(17:1)
Oleic acid	FFA(17:1)
Linoleic acid	FFA(17:1)

## Validation of protocol

As the lipid analysis on amide columns is widely used, several papers have sought to validate lipidomics methods using similar systems (Contrepois et al., 2018; Cao et al., 2019; Loef et al., 2020). For example, Medina et al. (2022) employed an amide column and ammonium acetate buffers in acetonitrile and water for their assay on plasma (Medina et al., 2022). Good linearity (r^2^ > 0.99) was obtained over broad concentrations, precision was determined by PCA plots of standards, and both intra-day and inter-day repeatability of < 25% were achieved for the majority of the lipids detected in the sample. Another study with similar parameters (Munjoma et al., 2022) obtained reproducibility coefficient of variance (CV) of < 1.0%, intra-day accuracy of ±20% CV of the nominal value, and less than 20% for intra-day precision for most of the lipids (Munjoma et al., 2022).

## General notes and troubleshooting

Troubleshooting is a complex process for analytical work using mass spectrometers. Common root causes for issues encountered in LC-MS include:

1. Clogging of column/in-line filter/transfer tubing;
2. Leakage in LC instrument;
3. Stationary phase collapse;
4. Contamination of ion-source/MS detector by sample molecules;
5. Signal suppression due to matrix effects.

The recommended corrective actions to address these issues have been outlined on the vendor’s website and other online resources (Steed, 2018; Agilent, 2023; Schug and Watson, 2023). The internal standard mixture or specific lipid standards can be used for troubleshooting purposes for our lipidomics method.

To isolate the underlying problem, it is advisable to follow a step-by-step approach: first, determine whether the issue is caused by LC or MS instrument, and then narrow the issue to specific LC or MS components. Usually, LC-related issues may manifest as alterations in retention time and peak shapes. On the other hand, MS-related issues may affect signal intensity and precision. A good way to distinguish which instrument is faulty is to run either a MS-based method (i.e., direct-injection into the mass spectrometer) or a LC-based method (i.e., using UV-detector) and see whether the issue still persists when one system is bypassed.

If we find that the issue lies with the LC system, it is useful to compare the pressure trace with past records and check whether it is too high or too low. High column backpressure may be indicative of a blockage (i.e., blocked column, plugged inlet-frit, clogged tubing). Low column backpressure is likely due to leakage (i.e., loose fitting, wear and tear of LC components). We can then proceed with the corrective action by starting from the most downstream component and working our way backward. For example, if we suspect there is a blockage, we can:

1. Clean/replace the column;
2. Replace the inlet-frit;
3. Replace tubing;
4. Clean the autosampler;
5. Clean the injection loop;
6. Check for microbial growth in solvent bottles.

Another two key pieces of information are the peak shapes and retention time, which can also be affected by the common root causes that we have listed above. For example, leaks, plugs, and deposits of material in the column may affect flow rate and cause variable shifts in retention time. A partially blocked frit can cause peak splitting or peak tailing because a portion of the sample may take a longer route around the blockage and arrive at the column later than the rest of the sample. Stationary phase collapse often leads to drastic changes in peak shapes and retention time. Readers are encouraged to read up on the following reference to learn how to interpret defective peak shapes (Dolan, 2015).

With regards to the MS system, contaminants constitute a serious problem, because they can potentially reduce signal intensity and increase background noise. For example, detergents such as polyethylene glycol and plasticizers such as phthalate esters are known to interfere with the analytes of interest by causing ion suppression (Rardin, 2018). Therefore, it is important to refrain from using soap or detergent in washing the glassware and minimize the use of plastic containers/vials during LC-MS experimentation. It is also good practice to clean the ion source and front-end of the instrument regularly. Additionally, one should always divert the unretained potion of the gradient at the beginning of each run to the waste.

Another important concern related to MS is matrix effects, whereby the components of the sample matrix may interfere with the ionization of the analytes of interest. One way to ascertain the occurrence of ion suppression is by spiking the lipid standards in pure solvent or sample matrix and comparing their percentage recovery. Matrix effects may be mitigated by diluting the samples, increasing the length of LC separation, or using solid-phase extraction to clean up the samples prior to introducing them into the MS.

## Supplementary information

The following supporting information can be downloaded here:

1. Table A1
